# Aluminum with dispersed nanoparticles by laser additive manufacturing

**DOI:** 10.1038/s41467-019-12047-2

**Published:** 2019-09-11

**Authors:** Ting-Chiang Lin, Chezheng Cao, Maximilian Sokoluk, Lin Jiang, Xin Wang, Julie M. Schoenung, Enrique J. Lavernia, Xiaochun Li

**Affiliations:** 1 Department of Mechanical and Aerospace Engineering, University of California, Los Angeles, CA 90095 USA; 2 Department of Materials Science and Engineering, University of California, Los Angeles, CA 90095 USA; 3 Department of Chemical Engineering and Materials Science, University of California, Irvine, CA 96297 USA

**Keywords:** Nanoscale materials, Metals and alloys

## Abstract

While laser-printed metals do not tend to match the mechanical properties and thermal stability of conventionally-processed metals, incorporating and dispersing nanoparticles in them should enhance their performance. However, this remains difficult to do during laser additive manufacturing. Here, we show that aluminum reinforced by nanoparticles can be deposited layer-by-layer via laser melting of nanocomposite powders, which enhance the laser absorption by almost one order of magnitude compared to pure aluminum powders. The laser printed nanocomposite delivers a yield strength of up to 1000 MPa, plasticity over 10%, and Young’s modulus of approximately 200 GPa, offering one of the highest specific Young’s modulus and specific yield strengths among structural metals, as well as an improved specific strength and thermal stability up to 400 °C compared to other aluminum-based materials. The improved performance is attributed to a high density of well-dispersed nanoparticles, strong interfacial bonding between nanoparticles and Al matrix, and ultrafine grain sizes.

## Abstract (web summary)

Incorporating and dispersing dense nanoparticles into metals remains a challenge. Here, the authors use nanocomposite powders containing very dense nanoparticles to print an aluminium nanocomposite with one of the highest specific modulus and yield strength among all structural materials.

## Introduction

Strong lightweight materials are an integral component of most energy and environmental sustainability strategies that are the hallmark of modern society^1,2^. Dispersed nanoparticles can be used to reinforce light metals^3–6^ while also refining grains and preventing solidification cracking^7,8^. Recently, laser additive manufacturing (LAM), or 3D printing, has emerged as a potent platform to accelerate materials innovation^9^ for aerospace, defense, automotive and biomedical industries. While LAM has been explored to process nanoparticles reinforced metals^10–12^, laser printed metals remain disadvantaged as far as their specific property improvements and thermal stability relative to those of other structural metals. A high density of nanoparticles in metals would further enhance their specific strength, specific modulus, and thermal stability. However, there are key challenges that hinder the achievement of an effective incorporation and dispersion of populous nanoparticles during LAM^13^.

It has been a long-standing problem to produce aluminum matrix nanocomposites (AMNCs) with a high density of dispersed nanoparticles. A molten salt assisted method has been reasonably effective to incorporate nanoparticles into bulk aluminum melts. Unfortunately when the loading of nanoparticles exceeds 10 vol. %, the contamination of molten salt into the aluminum melt poses as a serious problem, partly due to the higher viscosity of the nanocomposite melt^14,15^. Thus, it is not feasible to produce aluminum powders with dense dispersed nanoparticles by gas atomization of bulk AMNCs so far. On the other hand, it was demonstrated that a high loading of TiCN nanoparticles can penetrate micro Al droplets, without molten salt entrapment, to produce aluminum powders with dense nanoparticles^16^. However, a direct consolidation (casting or sintering) of these powders failed to produce high-quality AMNCs due to the dense nanoparticles coated on the powder surface, casting serious doubts in the feasibility of using these aluminum powders for LAM. Moreover, TiCN nanoparticles would react with super-heated aluminum melt during LAM.

Here we show that aluminum reinforced by dense dispersed nanoparticles can be deposited layer by layer via laser melting of aluminum nanocomposite powders, which enhances the laser absorption by almost one order of magnitude than pure aluminum powders. The laser printed nanocomposite delivers a yield strength of up to 1000 MPa, plasticity over 10%, and Young’s modulus of approximately 200 GPa, offering the highest specific Young’s modulus and one of the best specific yield strengths among all structural metals, as well as the best specific strength and excellent thermal stability at 400 °C amongst all aluminum-based materials. The high performance is attributed to a high density of well-dispersed nanoparticles, strong interfacial bonding between nanoparticles and Al matrix, and ultrafine grain sizes (about 331 nm). The pathway for laser 3D printing of nanoparticles reinforced aluminum can be readily extended to other materials to further break property limits for widespread applications.

## Results

### AMNC powders

**Fig. 1:**
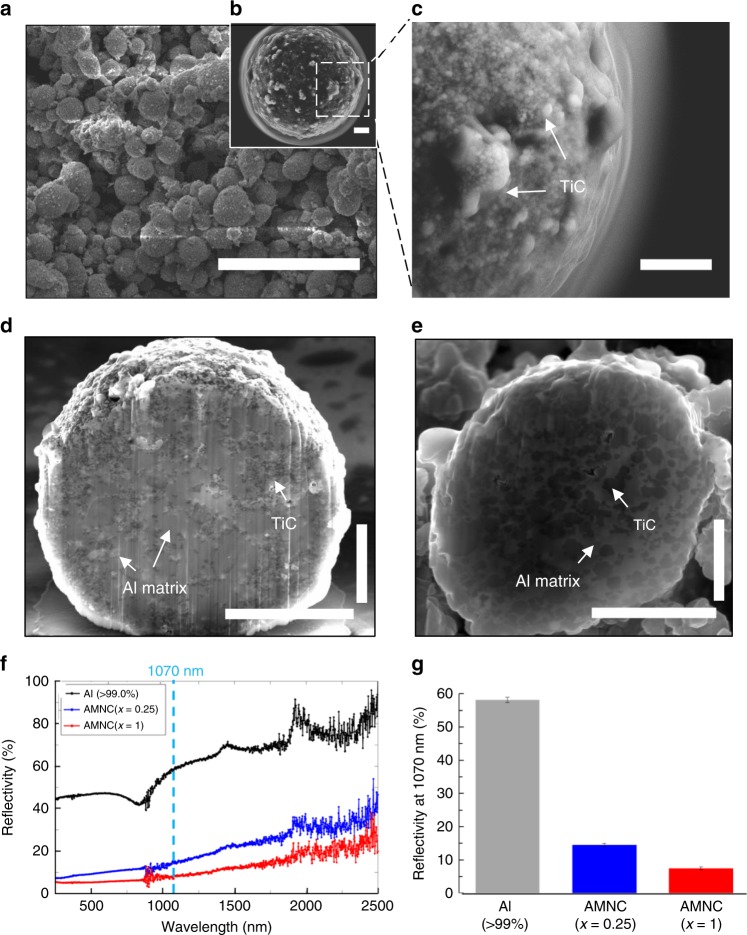
Morphology, microstructure and reflectivity of aluminum matrix nanocomposite (AMNC) powders. **a**, **b** SEM images of AMNC powders with *x* = 0.25. **c** Magnified image of **b**, showing TiC nanoparticles coated on the surface of an Al micro particle. **d**, **e** SEM images of the cross sections of specimens with *x* = 0.25 and *x* = 1, respectively. The AMNC powders (*x* = 1) have a higher loading of embedded TiC nanoparticles than the AMNC powders (*x* = 0.25). **f** Reflectivity of aluminum powders without and with nanoparticles. The dashed vertical line indicates the laser wavelength at 1070 nm. **g** Comparison of the reflectivity at the wavelength of 1070 nm for aluminum powder specimens with and without nanoparticles. Scale bar, 40 µm in **a**, 1 µm in **b**, **c**, **e**, and 4 µm in **d**. The error bars in the measured reflectivity are the standard deviations (s.d.) calculated over three measurements

We overcame significant challenges to design and fabricate aluminum powders that contain surface-coated and embedded nanoparticles suitable for LAM experiments. Here we demonstrate that laser-deposited aluminum that contains a high density of dispersed titanium carbide (TiC) nanoparticles (up to 35 vol.%) can be achieved. TiC was selected due to its chemical stability above 780 °C in aluminum melt (with a surprising twist that TiC is not chemically stable under 780 °C^17^. Aluminum matrix nanocomposite (AMNC) powders with dense TiC nanoparticles were systematically fabricated (see Methods). By simply tuning *x*, the volume ratio between TiC nanoparticles and liquid aluminum, AMNC powders with different TiC loadings (e.g., *x* = 0.25; and *x* = 1) can be fabricated (Also see chemical composition in Supplementary Fig. 1). As shown in Figs.1a and b, the AMNC powders (*x* = 0.25 and *x* = 1) are spherical with an average size of 11.3 ± 7.2 µm and 5.9 ± 4.6 µm, respectively (Supplementary Fig. 2). Most of the TiC nanoparticles would first assemble at the surface of the Al droplets, as shown in Fig. 1c (also see Supplementary Fig. 2b), to achieve a favorable energy state. As *x* increases, the favorable energy state is not available for the additional TiC nanoparticles; hence the nanoparticles are forced to enter the Al droplets. We conducted the experiment at 820 °C, at which the TiC is chemical stable with Al^17^ and the wetting angle of TiC/Al is less than 70°^18^, using ultrasonic processing (See Fabrication of AMNC powders in Methods**)**. The cross-section images in Fig. 1d and Fig. 1e indicate that the TiC nanoparticles (shown as gray spots) were also effectively pushed into and distributed inside the core of the Al powders. Spherical particles enable a better flowability and a higher packing density than irregularly shaped particles for LAM^19^. The surface-coated and uniformly dispersed TiC nanoparticles inside the AMNC powders can enhance laser beam absorption because of the high absorptivity of non-oxide ceramic nanoparticles^20,21^. The reflectivity measurements, as shown in Fig. 1f and Fig. 1g, illustrate that the reflectivity of AMNC powders with *x* = 0.25 and *x* = 1 are 14.58 ± 0.46% and 7.46 ± 0.47%, respectively, at the laser wavelength of 1070 nm for the LAM process in this study. The reflectivity of AMNC powders is thus significantly lower than that of pure aluminum powders (58.12 ± 0.81%), which enhances the laser absorption by almost one order of magnitude. A comparison between theoretical values and experimentally measured reflectivity (Supplementary Fig. 3) shows that the actual reflectivity (*x* = 0.25; *x* = 1) are much lower, i.e., 70% and 80%, than the predictions by the equation. It could be attributed to the multiple reflection and absorption that is inherent to the porous structure of the powder bed and the fact that the concentration of TiC nanoparticles on the nanocomposite powder surface is significantly higher than overall concentration.

### Structures of laser-deposited AMNCs

**Fig. 2:**
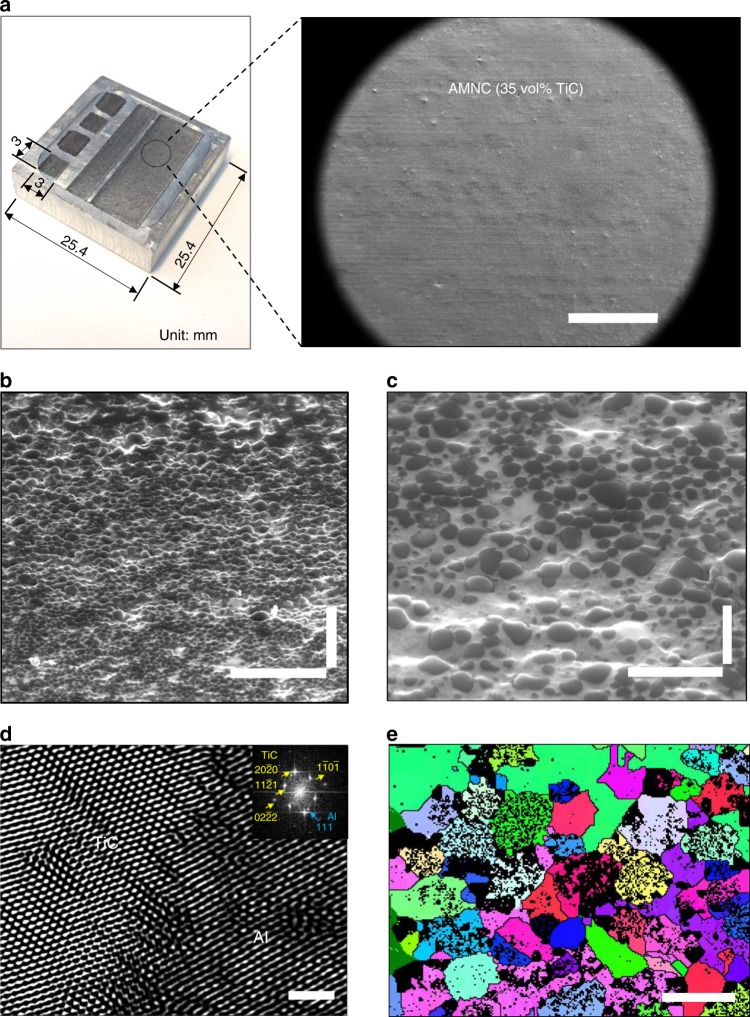
Surface and micro/nanostructure of laser-deposited aluminum matrix nanocomposites (AMNC). **a** Top view of laser-deposited AMNC (35 vol.% TiC) specimens with dimensions of 3 mm × 3 mm, 3 mm × 18.5 mm, and 8 mm × 18.5 mm. The insert SEM image shows an area of the laser-deposited specimen. **b**, **c** 52° tilted cross-sectional SEM images of laser-processed AMNC (35 vol.% TiC) were captured under different magnifications, showing that TiC nanoparticles are uniformly dispersed and distributed in aluminum. **d** FFT filtered high resolution TEM image shows good bonding between TiC nanoparticle and aluminum. Inserts are the fast Fourier transforms corresponding to the planes of (2 2 0) aluminum matrix and of (2 0 0) TiC nanoparticles. e, The grain maps of laser-deposited AMNC (35 vol.% TiC). Scale bar, 1 mm in a (right), 5 µm in **b**, 1 µm in **c**, 2 nm in d, and 500 nm in **e**

To obtain structures of laser-deposited AMNCs, extensive experiments were conducted on LAM of AMNC powders (see Methods). Then we characterized the micro/nanostructure and mechanical properties of the laser-deposited AMNC. Figure 2a shows the AMNC with 35 vol.% TiC with a thickness of 309 ± 16 µm was layer by layer deposited by laser melting of the Al powder (*x* = 1) bed, which was preheated to 300 °C. A SEM image was captured from the top of the deposited specimen (8 mm × 18.5 mm), showing the AMNC has a good uniformity and is well bonded to the previous layers. To reveal the interior microstructure, the polished AMNC specimen was tilted 52° to acquire cross-sectional images (See Microstructure characterization in Methods), as shown in Fig. 2b and Fig. 2c, indicating that a high volume fraction of TiC nanoparticles was dispersed and distributed homogeneously throughout the Al matrix. The uniform dispersion and distribution of TiC nanoparticles in Al matrix can be attributed to the unique nature of laser processing and the good wetting of TiC in molten Al. The laser-induced rapid cooling rate can reach up to 10^6–7^ K s^−1^ ^22^, and therefore the movement of atoms and particles freezes within milliseconds. The initial AMNC powders were fully melted and then solidified rapidly. During the non-equilibrium laser-induced rapid melting and solidification, both the TiC nanoparticles at the surface and inside of AMNC powders did not agglomerate to form clusters. Despite the formation of some larger particles with an average size of 159 nm (as shown by TEM in Supplementary Fig. 4) after solidification, TiC nanoparticles were still uniformly dispersed and distributed in AMNC specimens, as shown in the cross-sectional image of Fig. 2b, c. Moreover, TiC nanoparticles bond with Al matrix extremely well as confirmed in the FFT filtered high resolution TEM image (Fig. 2d). The TiC nanoparticles on the powder surface can absorb the laser beam more effectively to achieve a much higher temperature relative to the melting point of aluminum, enabling a rapid dispersion and diffusion of surface TiC nanoparticles into the core of the molten aluminum powders to expose liquid aluminum for bonding into dense layers. The rapid heating and cooling during the LAM process also limited the chemical reaction of TiC below 780 °C in aluminum melt.

It is argued that the high density of uniformly distributed TiC nanoparticles plays a critical role of refining the Al grains because the nanoparticles can act as nucleation sites and also restrict the growth of the Al grains during solidification. The EBSD mapping results revealed the grain size and crystallographic texture difference from the laser-deposited specimens of pure Al and AMNC (35 vol.% TiC), as shown in Supplementary Fig. 5a, 5b and Fig. 2e. Clearly, TiC particles, i.e., the black spots shown in Fig. 2e, were uniformly distributed in the Al matrix grain, indicating that TiC nanoparticles were well dispersed and distributed throughout the Al matrix. For the high (i.e., 35 vol.%) volume fraction of reinforcing TiC nanoparticles and the good dispersion of these nanoparticles, it is necessary to remove the TiC phase from Fig. 2e to better reveal the grain size of the refined Al matrix (see further details in Supplementary Fig. 5). Whereas the average grain size for the pure aluminum is approximately 2.7 ± 1.4 µm, the average grain size for the AMNC (35 vol.% TiC) was refined to 331 ± 95 nm (see further details in Supplementary Fig. 5). Recent studies also observed that the grain size of the laser additive Al specimen can be reduced after the incorporation of ceramic nanoparticles^20,23^. The TiC nanoparticles promote grain refinement via two mechanisms. First, they provide a high density of nucleation sites, leading to a finer grain size once the liquid solidifies. Second, the TiC nanoparticles impede migration of the newly formed grains, thereby stabilizing the grain size. The TiC nanoparticles can be used as grain growth inhibitors in Al as they can be treated as pinning points, inhibiting the grain growth during solidification, recrystallization, and recovery. The phenomenon has been reported in other Al-TiC nanocomposites^24^, showing that a high volume fraction of fine particles is very effective for grain growth retardation. In addition, the rapid cooling rate from laser processing can further contribute to the small grain size. Thus, the decrease of grain size can be attributed to a combined effect of augmented nucleation sites, restricted grain growth and high cooling rate during LAM.

### Mechanical properties of AMNCs

**Fig. 3:**
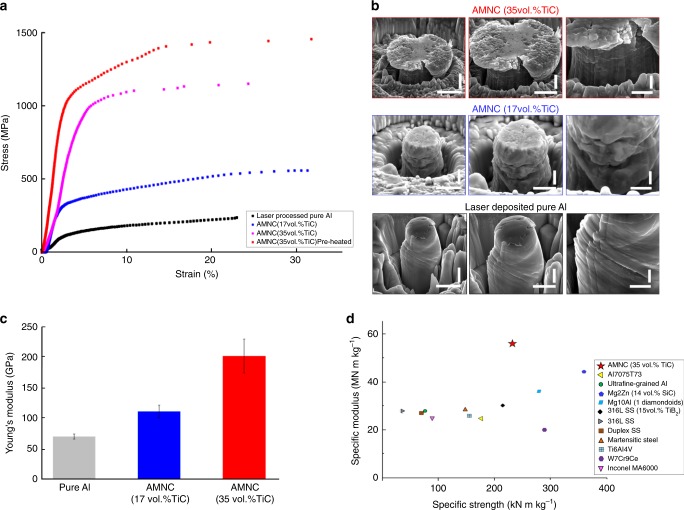
Room-temperature mechanical behavior of laser-deposited aluminum with and without nanoparticles. **a** Typical engineering stress-strain curves of laser-deposited Al specimens with and without nanoparticles. **b** SEM images of micropillars after microcompression tests. **c** Young’s modulus of laser-deposited Al and AMNC specimens. Error bars represent SD for at least twenty data sets. **d** Specific Young’s modulus and specific yield strength of AMNC and other materials (all data from microcompression tests without size effect). Scale bar, 3 µm (left), 2 µm(middle) and 1 µm(right) in **b**

To evaluate the enhancement of mechanical properties due to such a high density of TiC nanoparticles, we first conducted microcompression tests at room temperature. Micropillars with a diameter of 4.0 ± 0.1 µm and a height of 10.0 ± 0.5 µm were carefully machined by FIB from the laser-deposited specimens with and without nanoparticle reinforcements. It should be noted that the locations of the micropillars were chosen randomly, and all testing data shown in this study were conducted more than 3 times. As shown in Fig. 3a, the pure aluminum specimen has a yield strength of only about 92 ± 16 MPa (Fig. 3a, the curve in black), while the AMNC specimens (with 17 vol.% TiC, processed at 25 °C, i.e., no preheating) offer a yield strength of up to 300 ± 52 MPa (Fig. 3a, the curve in blue). With a higher TiC loading (35 vol.%), the yield strength of the as-deposited AMNC reaches 868 ± 104 MPa with a plasticity greater than 10%, as shown in the curve in purple in Fig. 3a. Data for each curve was obtained by at least three sets of experiments. To improve the layer uniformity during laser melting, the powder layers were pre-heated at 300 °C. The result shows that the yield strength of AMNC (35 vol.% TiC, pre-heated) is about 906 ± 105 MPa (Fig. 3a the curve in red) with a plasticity greater than 10%, slightly higher than that of result without preheating the powder bed. The compression performance is expected to improve because of the different thermal gradients. Specifically, the preheating AMNC powder bed can avoid solidification cracking since the cooling rate is affected^25^. This can result in the improvement of residual stresses and distortion of the counterpart during the layer-by-layer process^26^. We then characterized the micropillar deformation after the compression test, as shown in Fig. 3b. Multiple slip bands appeared in the laser-deposited pure aluminum specimens, which are common for microcompression tests of face-centered cubic micropillars^27^. In contrast, the AMNC specimens have significantly fewer slip bands as compared to those in the pure Al specimens. It is highly likely that TiC nanoparticles in the Al specimens can sustain higher compression loads, resulting in significant higher yield strength. This hypothesis can also be validated by the compression test at the elevated temperature of 400 °C (See Supplementary Movie 1 and Supplementary Fig. 6).

To understand the strength obtained in as-deposited AMNCs, the strengthening mechanisms for the AMNC (35 vol.% TiC) can be possibly attributed to Orowan strengthening^28^, Hall-Petch effect^29^, and load-bearing transfer, which are estimated to be approximately 294, 104, and 525 MPa, respectively (see Mechanical Strengthening Mechanisms in Methods). The strong interfacial bonding as shown in Fig. 2d suggests that its theoretical value would be approximately 1000 MPa with a load-bearing transfer strengthening of 525 MPa. However, it should be noted that there is no direct evidence indicating interfacial bonding strength.

It is postulated that the good interfacial bonding between the nanoparticles and Al matrix results in the superior elastic modulus in the AMNC specimens. Figure 3c shows that the Young’s modulus of the laser-deposited AMNC specimens is significantly enhanced when compared to that of pure aluminum. While the pure aluminum specimen has a Young’s modulus of 68 ± 4 GPa in our tests, the AMNC (17 vol.% TiC) and AMNC (35 vol.% TiC) specimens offer a Young’s modulus of 108 ± 10 GPa and 197 ± 27 GPa, respectively. Figure 3d shows the specific Young’s modulus and specific yield strength of the AMNC specimens and other engineering alloys, indicating that the AMNC (35 vol.% TiC) exhibits the highest specific Young’s modulus and one of the best specific yield strengths among all structural metals (see Comparison of specific mechanical properties with other materials in Methods). An extensive review of conventional aluminum matrix composites (AMCs), i.e. especially aluminum reinforced with TiC micro particles, is included in Supplementary Table 1. All AMC-TiC composites offer much lower mechanical properties and Young’s modulus than our laser printed AMNCs (See Supplementary Table 1). The comparison of the specific modulus and yield strength between the AMNCs and any other aluminum-based materials (aluminum alloys and composites) is shown in Supplementary Fig. 7. While the microcompression tests conducted using micropillars without size effect provide scientifically-meaningful yield strength values, Young’s modulus, and uniform plasticity to characterize the laser printed AMNCs, tensile testing would pose a more serious challenge for ductility in the laser-printed specimens, which heavily depends on engineering optimization. It should be noted, however, that uniform plasticity of about 10% in the laser-printed AMNCs is a good indication that the material can withstand some plastic deformation. It is worth noting, however, that in the case of high-temperature applications, ductility may not be an issue.

### High temperature stability

**Fig. 4:**
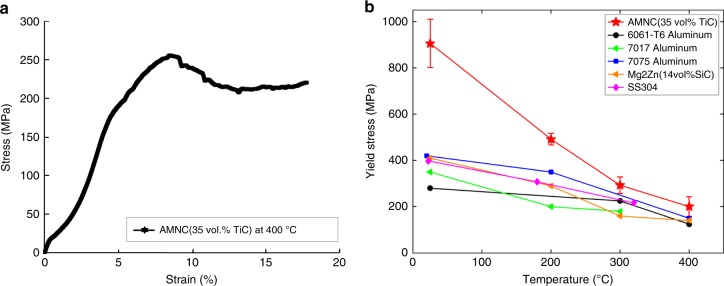
Mechanical behavior of laser-deposited AMNC at elevated temperature. **a** Typical engineering stress–strain curves for microcompression of laser-deposited AMNC (35 vol.% TiC) at 400 °C. **b** Yield strength of AMNC (35 vol.% TiC) at test temperatures of 25, 200, and 400 °C in comparison with other materials. Error bars show s.d. of three tested samples

It is well known that most aluminum alloys lose their strength at elevated temperatures due to the rapid coarsening of grain size and loss of strengthening precipitates^30^. To evaluate the high-temperature stability of the laser-deposited AMNC (35 vol.% TiC) specimens, microcompression tests were conducted at 200, 300, and 400 °C (See in-situ microcompression test at elevated temperatures in Methods). The results from the in-situ microcompression (See Supplementary Movie 1) after testing reveal that the AMNC specimens can still reach a yield strength of 200 ± 43 MPa with a plasticity greater than 15% at 400 °C, as shown in Fig. 4a. To understand the strength contribution, the microstructure of 400 °C tested specimen was studied in detail (See Supplementary Fig. 8a), revealing that the fine particles are still well dispersed and distributed. Since the average particle diameter and the grain size are expected to change under high-temperature testing condition, the estimated strengthening from Hall-Petch effect and Orowan strengthening are ~77 and 25 MPa, respectively (See detailed in Supplementary Fig. 7). The strong interfacial bonding is still very likely the main strengthening contribution, which is a promising direction for further study to validate the hypothesis. Moreover, to further determine the thermal stability after a heating period of 1.0 h at 400 °C, the AMNC specimens were cooled down to room temperature and again tested using microcompression testing at 25 °C (See High-temperature stability measurement in Methods). The results show that the AMNC (35 vol.% TiC) specimens still exhibit a yield strength greater than 800 MPa with a plasticity greater than 10%. Figure 4b also shows the AMNC (35 vol.% TiC) specimens offer exceptional strength when compared with other engineering materials at different elevated temperatures. At 400 °C, the AMNC specimens offer a higher strength than any other aluminum materials and even greater than stainless steel SS304. These results clearly suggest that AMNC with dispersed nanoparticles deposited via laser additive manufacturing, not only exhibits yield strength and plasticity that are superior to those of previously reported Al-based materials but also provides exceptional high-temperature stability (see Comparison of yield stress at elevated temperatures in Methods).

In summary, aluminum with dense dispersed nanoparticles was layer-deposited via laser additive manufacturing of AMNC powders, delivering the highest specific Young’s modulus and one of the best specific yield strengths among all structural metals, as well as an thermal stability at 400 °C amongst all aluminum-based materials. The AMNC powders allow a higher laser absorption by almost one order of magnitude than the pure aluminum powders. The pathway for laser 3D printing of nanoparticles reinforced metals can be readily extended to other materials for widespread applications.

## Methods

### Fabrication of AMNC powders

To produce the AMNC powders, premixing of NaCl and KCl salt powders (from Fisher Chemical, ≥99.0%), TiC nanoparticles (with an average size of 40–60 nm, from US Research Nanomaterials, ≥99.0%) and Al microparticles (with average size of 20 µm, from Sigma-Aldrich, ≥99.0%) was carried out in a mechanical shaker for 30 mins. Then, the mixture was dehydrated in a vacuum furnace at 120 °C for 10 mins. The mixture was melted at 820 °C under argon protection in a graphite crucible with an outside diameter of 58 mm and a height of 88 mm. An ultrasonic niobium probe with a diameter of 12.7 mm and a length of 92 mm was then inserted 6 mm deep into the molten liquid, processing the melt for 15 mins before the specimens were taken out of the furnace and cooled down in air environment. The cooled specimens were then repeatedly dissolved four times in 400 mL distilled water in an ultrasonic bath for 30 mins. The solution was filtered through filter papers with a mesh size of 2.7 µm (Whatman plc) using vacuum filtration under room temperature for 20 mins. Eventually the AMNC powders were obtained and collected from the top of the filter papers. These powders were dried and dehydrated in a vacuum furnace for 10 mins at 150 °C before their use in laser experiments. To control the nanoparticle loading in the AMNC powders, we tuned the volume ratio, x, of the TiC nanoparticles to the Al microparticles. The volume ratio between the powder mixture and the salt was maintained constant at 3%. For the AMNC powder with a volume ratio factor of x = 0.25, we mixed 1.57 g TiC nanoparticles, 3.6 g Al micro particles, 27.1 g NaCl, and 34.6 g KCl. For AMNC powder with a volume ratio factor of *x* = 1, 4.39 g TiC nanoparticles were mixed 2.4 g Al, 27.1 g NaCl, and 34.6 g KCl. Ultrasonic amplitude of 30 µm and 45 µm were used for processing of the materials with *x* = 0.25 and *x* = 1, respectively.

### Laser additive manufacturing of pure aluminum and AMNC

The schematic of a customized laser additive manufacturing system is illustrated in Supplementary Fig. 9. The experiment was conducted by a 1070 nm fiber laser (SP-200C-W-S6-A-B, SPI Lasers) tuned to a power output of 200 W, a scan speed of 0.2 m/s at continuous wave mode, a spot size of 50 µm, a hatching space of 30 µm, and a 90° scanning direction difference for each layer, as well as a customized stainless vacuum chamber (with a vacuum level at about 1 × 10^−2^ torrs). A temperature control system was installed for preheating of the powders. For each cycle of laser deposition, a AMNC powder layer (*x* = 0.25 and *x* = 1) with a thickness of about 50 µm was manually deposited on a pre-machined Al 1100 alloy substrate (≥99.0%, McMaster-Carr) with a dimension of 25.4 mm × 25.4 mm × 6.27 mm. The thickness of the powder layer was guided by a customized layer-thickness control device. A *z*-axis manual stage was placed in the chamber to manually adjust the laser focal point since the height of a new layer of powders will be different after laser melting of each layer. After the specimens were mounted onto the temperature control system inside the vacuum chamber, it was firstly evacuated to a vacuum level of about 1 × 10^−2^ torr, followed by a constant argon flow about 30 mins to reduce the oxygen content in the working environment. The argon purging process, i.e., vacuum pumping followed by argon purging, was repeated twice before of the preheating and laser selective melting. The laser scanning patterns of 3 mm × 3 mm, 3 mm × 18.5 mm, and 8 mm × 18.5 mm were processed the powder layers at temperatures of 25 °C (without preheating) or 300 °C (with preheating). The layer deposition process was repeated to obtain a designed layer thickness for fundamental study. In this study, the AMNC specimens with a thickness of 100 ± 16 µm (AMNC with17 vol.% TiC) and of 309 ± 16 µm (AMNC with 35 vol.% TiC) were deposited for characterization. Laser-processed pure Al specimens with a thickness of about 192 ± 30 µm were also obtained for comparison.

### Powder characterization

Light scattering (LS) particle analyzer (LS13 320, Beckman Coulter) was used to determine the size distribution of the AMNC powders (Supplementary Fig. 2c and Fig. 2d). Scanning Electron Microscopy (SEM, Supra 40VP, ZEISS) and Focused Ion Beam (FIB, Nova 600, FEI) were utilized to study the surface and the inner microstructures of the spherical AMNC powders. To reveal the distribution and dispersion of TiC nanoparticles in the AMNC powders, the specimens were mounted on a silicon wafer, tilted to 52° and then etched by FIB with gallium ions. The images of the 52° tilted cross-sectional SEM powders were acquired to reveal the distribution and dispersion of the nanoparticles. EDS (Energy-dispersive X-ray spectroscopy) with a mapping scan mode was used to characterize the chemical compositions of the AMNC powders. At least 40,000 elemental signals, i.e., net counts, were captured to determine the elemental compositions.

A UV3101PC spectrophotometer (SHIMADZU Cop.) was used to measure the reflectivity of the aluminum powder specimens without (i.e., Al ≥ 99.0%) and with the reinforcement nanoparticles (i.e., 17 vol.% and 35 vol.% of TiC nanoparticles). A wavelength range from 250 nm to 2500 nm was scanned using UV/visible/NIR detectors (photomultiplier and PbS cell) with a spectral resolution of 2.0 nm. A standard barium sulfate BaSO_4_ plate was used to perform a baseline correction over the required wavelength range to ensure a 100% reflectance. Powder specimens were then mounted and sealed on a powder specimen holder packed with the accessory barium sulfate BaSO_4_ for the reflectance measurement. The kinetic measurement mode was applied to record and analyze the reflectivity using a multifunctional UVProbe software (SHIMADZU Cop.).

### Microstructure of laser layer-deposited AMNC specimens

SEM and TEM were used to reveal the dispersion and distribution of the TiC nanoparticles in the laser-processed AMNC specimens. The specimens were first vertically mounted in epoxy holders with an outside diameter of 30 mm, and then filled with a mixture of a transparent curable epoxy and a hardener (Allied High Tech Products, Inc.) with a ratio of 10–3, followed by grinding and polishing. To clearly expose the TiC nanoparticles on the surface of the Al matrix, polished specimens were 52° tilted and slightly etched by FIB with gallium ions, followed by obtaining SEM images with EDS (Energy-dispersive X-ray spectroscopy) analysis. High resolution TEM images were obtained by Scanning/Transmission Electron Microscopy (S/TEM, Titan, FEI). The TEM specimens prepared by FIB were obtained from the micropillars after compression tests (Supplementary Fig. 4a). Fourier-filtered high resolution TEM images were obtained to reveal the interface between the TiC nanoparticles and the Al matrix.

To reveal the grain size of laser-deposited specimens with and without nanoparticles, Electron backscatter diffraction (EBSD) was utilized to characterize the pure aluminum specimen while Transmission-EBSD was used to observe the FIB-prepared AMNC specimen (35 vol.% TiC) with a thickness less than 100 nm. Both specimens were placed in the SEM chamber, 70° tilted from horizontal towards the EBSD-diffraction camera. The EBSD/Transmission-EBSD scans were performed at a voltage of 20 and 30 kV and a current of 5.3 nA and 12 nA, respectively. The mapping scans were captured and evaluated by HKL Channel5 software. ImageJ software was used to further validate the grain size distribution as shown in Supplementary Fig. 5.

### Mechanical characterization

A MTS XP Nanoindenter was used to conduct microcompression tests to study the mechanical properties of the laser-processed specimens with and without TiC nanoparticles. FIB-machined micropillars with a size of 4.0 ± 0.1 µm in diameter and a height of 10 ± 0.5 µm were compressed by a flat punch probe with a size of 10 µm at room temperature using the displacement control mode, and a strain rate of 2 × 10^−3^ s^−1^.

In-situ microcompression tests test at elevated temperatures were conducted using a PI 95 PicoIndenter (Hysitron Inc.) with a flat punch diamond probe of 20 µm inside a FEI Quanta 3D SEM/FIB. FIB-machined micropillars (4 µm in diameter and 9 µm in height) from a AMNC (35 vol.% TiC, preheated at 300 °C) specimen were compressed using the load-control mode, and a strain rate of 2 × 10^−3^ s^−1^. The real time load-displacement data and in-situ deformation movies of micropillars were monitored, captured and recorded by TriboScan (Hysitron Inc.). In-situ microcompression was conducted at temperatures of 200, 300, and 400 °C by a resistive microelectromechanical systems (MEMS) heater. A AMNC (35 vol.% TiC, preheated at 300 °C) specimen was attached to the MEMS temperature-control specimen heater using high-temperature silver conductive epoxy (Ted Pella product #16014), followed by installing the specimen heater on the PicoIndenter system. The resistance of the heating element was utilized to elevate the specimen temperature to a desired value. Each temperature level was maintained 300 s before the compression tests. A resistive temperature detector (RTD) sensor was used to measure the real time temperature and provide feedback for the MEMS temperature controller.

Microcompression test of specimens after exposure at elevated temperatures were performed to further determine the thermal stability. AMNC (35 vol.% TiC) specimens, after a heating period of 1.0 h at 400 °C were cooled down to room temperature and further microcompression tests were conducted again under room temperature. Micropillars with a diameter of 4 µm and a height of 9 µm were prepared by FIB. All testing parameters and experimental setups remained the same as in the section of Microcompression test.

Measurements of elastic modulus were performed using nanoindentation tests by MTS Nanoindenter XP to evaluate the elastic modulus of the laser-processed materials. The specimens with nanoparticles (17 vol.% TiC and 35 vol.% TiC) and without nanoparticles (pure aluminum) were compressed by a Berkovich tip with an indentation depth of 2 µm. For each specimen, 20 randomly selected points were measured. Elastic moduli were calculated from the unloading curves.

### Comparison of specific mechanical properties with other materials

To compare the AMNC (35 vol.% TiC) specimens with other representative engineering alloys, all testing data were collected from micropillar compression tests without size effect. The diameters of testing specimens were in the range of 3.5–7 µm. It should be noted that the properties reviewed were obtained by using different strain rates and the data points shown in the graph were the extreme values data presented in that reference, i.e., the highest values for the yield strength. Also, since the authors^10,31^ did not provide exact Young’s modulus data, the data were estimated using a superposition method, which would theoretically be higher than the experimental data. The references for each material are listed as follows: Al7075 T73^32^, Ultrafine-grained Al^33^, Mg2Zn (14 vol.% SiC)^6^, Mg10Al (1 diamondoids)^34^, 316 L stainless steel, and 316 L stainless steel (15vol.% TiB_2_)^10^, Duplex stainless steel^35^, Ti6AI4V^36^, and W7Cr9Fe^37^ and Inconel MA6000^38^.

### Comparison of yield stress at elevated temperature

The yield stress at elevated temperatures is sensitive to different strain rates. It should be noted that a high strain rate typically leads to a strengthening effect. Therefore, to scientifically compare our AMNC (35 vol.% TiC) specimens with other representative engineering alloys, we also listed the strain rate data here: a strain rate of 1 × 10^−2^ for aluminum alloys; of 2 × 10^−3^ for current strongest magnesium nanocomposite, and of 1 × 10^−4^ for SS304. The test of SS304 was conducted according to the ASTM Standard E21–92. For each material, the references are listed as follows: 7017 Aluminum^39^, SS304^40^, Mg_2_Zn (14 vol.% SiC)^6^, 7075 Aluminum^41^, and 6061 Aluminum^42^.

### Mechanical strengthening mechanisms

To understand the strength obtained in as-deposited AMNC, the strengthening mechanisms for the AMNC (35 vol.% TiC) can be possibly attributed to Orowan strengthening^28^, Hall-Petch effect^29^, and load-bearing transfer. Here we discuss these potential strengthening contributions in the laser-deposited AMNC (35 vol.% TiC) specimens.

The contribution from Orowan strengthening can be estimated by^28^:

1 $${\Delta \sigma _ {\mathrm{Orowan}}} \,=\, {0.13 {\mathrm{G}}_{\mathrm{m}}{\mathrm{b}} \ast ln\left( {\mathrm{r}/{\mathrm{b}}} \right)/\lambda}$$

2 $$\lambda \approx {\mathrm{d}}_{\mathrm{p}} \ast [(1/2 {\mathrm{V}}_{\mathrm{p}})^{1/3} - 1]$$

where r is the particle radius, and *λ* is the inter-particle spacing, d_p_ is the particle diameter, b is the Burger’s vector, V_p_ is the volume fraction of nanoparticles, and G_m_ is the matrix shear modulus. The values for the AMNC specimens with 35 vol.% TiC are: b = 0.286 nm, G_m_ = 25.5 GPa, V_p_ = 0.35, r = 0.5 and d_p_ = 79.5 nm. The particle size is determined by the TEM results in Supplementary Fig. 3. The$\Delta \sigma _{Orowan}$is thus determined to be ~294 MPa.

It is well known that the grain size has a significant influence on metal yield strength since grain boundaries act as obstacles for dislocation movement. The TiC nanoparticles can serve as nucleation sites and also the pinning points to inhibit the Al grain growth during solidification. From our EBSD mapping results, the average grain size of the laser-deposited AMNC (35 vol.% TiC) specimen is approximately 331 ± 95 nm, as shown in Fig. 2e (also see Supplementary Fig. 5). The yield strength gained from the Hall-Petch strengthening can be calculated by:

3 $$\Delta \sigma _ {\mathrm{H}\_\mathrm{P}} = {\mathrm{kd}}^{ - 1/2}$$

where k = 0.06 MPa m^1/2^ as the strengthening coefficient for aluminum^42^, as d the average grain size in the AMNC specimen. The calculated ∆σ_H_P_ is 104 MPa.

It is believed that the load-bearing effect significantly contributes to the strengthening of the AMNC specimen since the interfacial bonding between the nanoparticles and aluminum is excellent, as shown in as shown in Fig. 2d. A strong interfacial bonding and a dense homogeneous dispersion of TiC nanoparticles (see Supplementary Fig. 4b) can result in strengthening for the AMNC specimens, which can be estimated by^43^:

4 $$\Delta \sigma _ {\mathrm{load}} = 1.5 {\mathrm{V}}_{\mathrm{p}}\sigma _ {\mathrm{i}}$$

where *V*_p_ is the volume fraction of particles and σ_i_ is the interfacial bonding between Al matrix and TiC nanoparticles. The strong interfacial bonding as shown in Fig. 2d suggests that the theoretical value of σ_i_ would be ~1000 MPa (with a hypothetical ∆σ_load_ at 525 MPa). It should be noted that this is purely hypothesis as there is no interfacial bonding strength data available.

## Supplementary information

Supplementary information

Description of Additional Supplementary Files

Supplementary Movie 1

## Data Availability

All relevant dataset generated during and/or analyzed in current study are available from the authors.
