# Supplementary material for: Aluminum with dispersed nanoparticles by laser additive manufacturing
Source: Nat Commun. 2019 Sep 11;10:4124. doi: 10.1038/s41467-019-12047-2 (PMC6739343; doi:10.1038/s41467-019-12047-2)
Supplement: Supplementary file 1 — Supplementary information [file 41467_2019_12047_MOESM1_ESM.pdf]

Aluminum with Dispersed Nanoparticles by Laser Additive Manufacturing

Ting-Chiang Lin et al.

Supplementary Figure

a

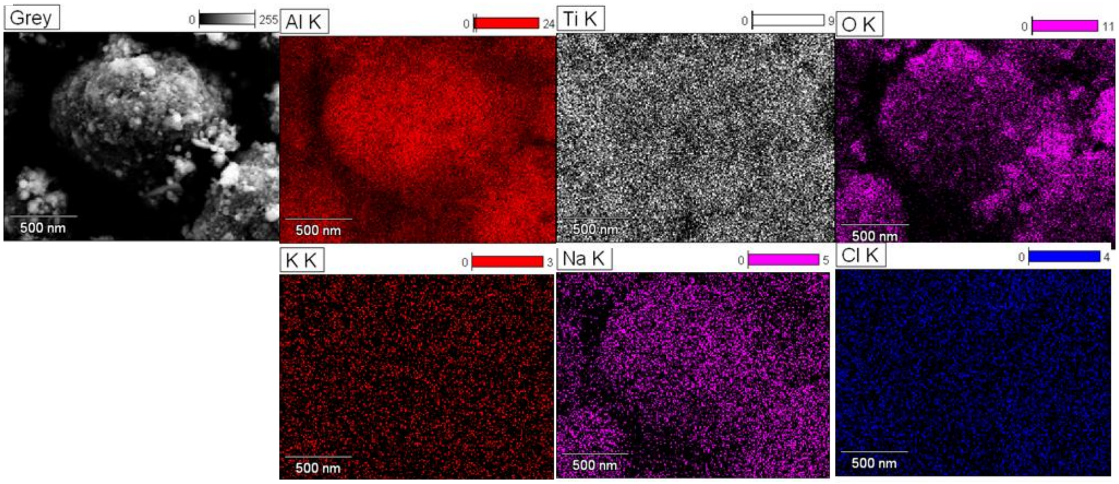

b

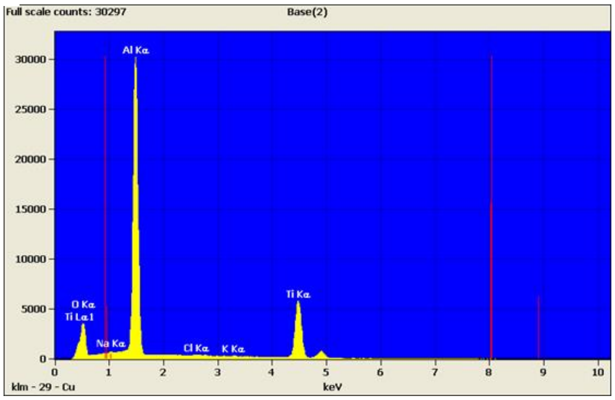

c

Element	Net Counts	Element Wt.%	Wt.% Error
O	18012	8.07	+/-0.16
Na	535	0.1	+/-0.02
Al	300617	37.37	+/-0.10
Cl	624	0.14	+/-0.03
K	556	0.16	+/-0.03
Ti	81796	54.17	+/-0.30
Total	402140	100	

Supplementary Figure 1 | A selected chemical composition analysis from AMNC powders (x=1). **a**, elemental composition. **b** and **c**, EDS spectrum signals and its corresponding elemental counts and weight percentage. The results reveal the powders were mainly composted of Al, Ti, and oxygen, and very minor K and Cl elements. It seems that the minor impurities did not affect the final property of the laser deposited aluminum, possibly the high laser processing temperature burn the surface contained K and Cl away effectively

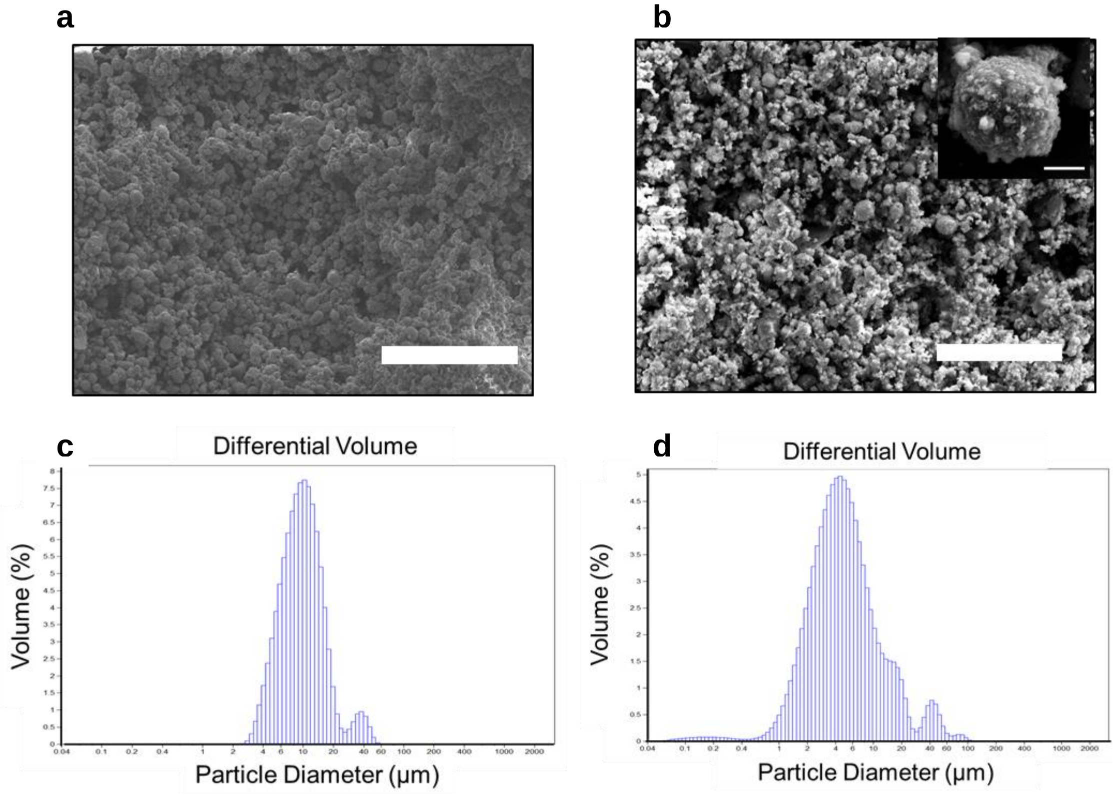

Supplementary Figure 2 | Characterization of AMNC powder. SEM images of AMNC powders from different volume ratios of **a**, *x*= 0.25 **b**, *x*= 1. **c**, **d**, Size distribution of AMNC powders for *x*=0.25 and *x*=1, respectively. Scale bar, 100 µm in a, 1 µm in b(insert) and 20µm in b.

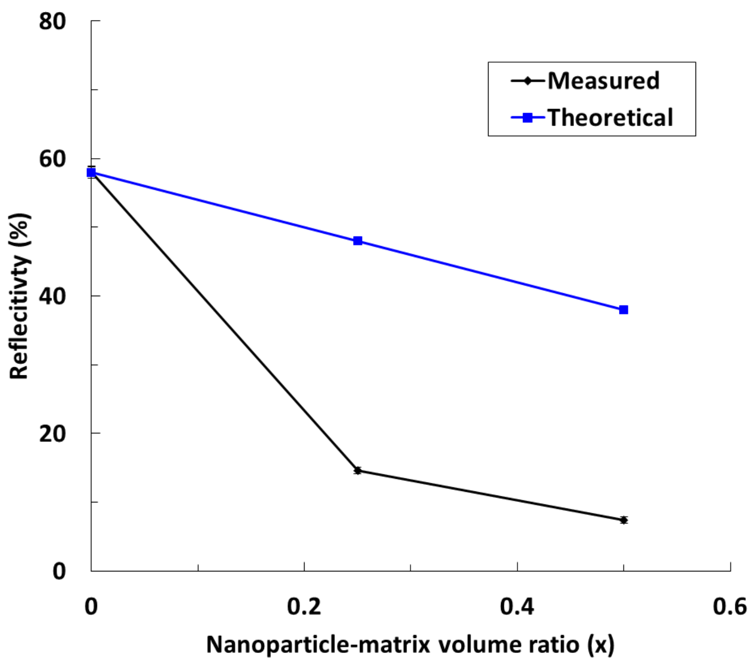

Supplementary Figure 3 | Comparison of the theoretical reflectivity with actual measured values. The predicted absorptivity of a powder mixture containing two components used for laser processing was calculated by a theoretical equation[1]: $A = A_1 r_1 + A_2 r_2$, where A_i is absorptivity and r_i is the volume fraction of the component. The absorptivity (Ai) of a material is defined as a ratio of the absorbed radiation to the incident radiation and can be calculated by $A = 1 - R$, where R is the measured reflectance in this study. The equation has been experimental validated with an accuracy of 10 percent, and can be used to estimate roughly to other material systems.

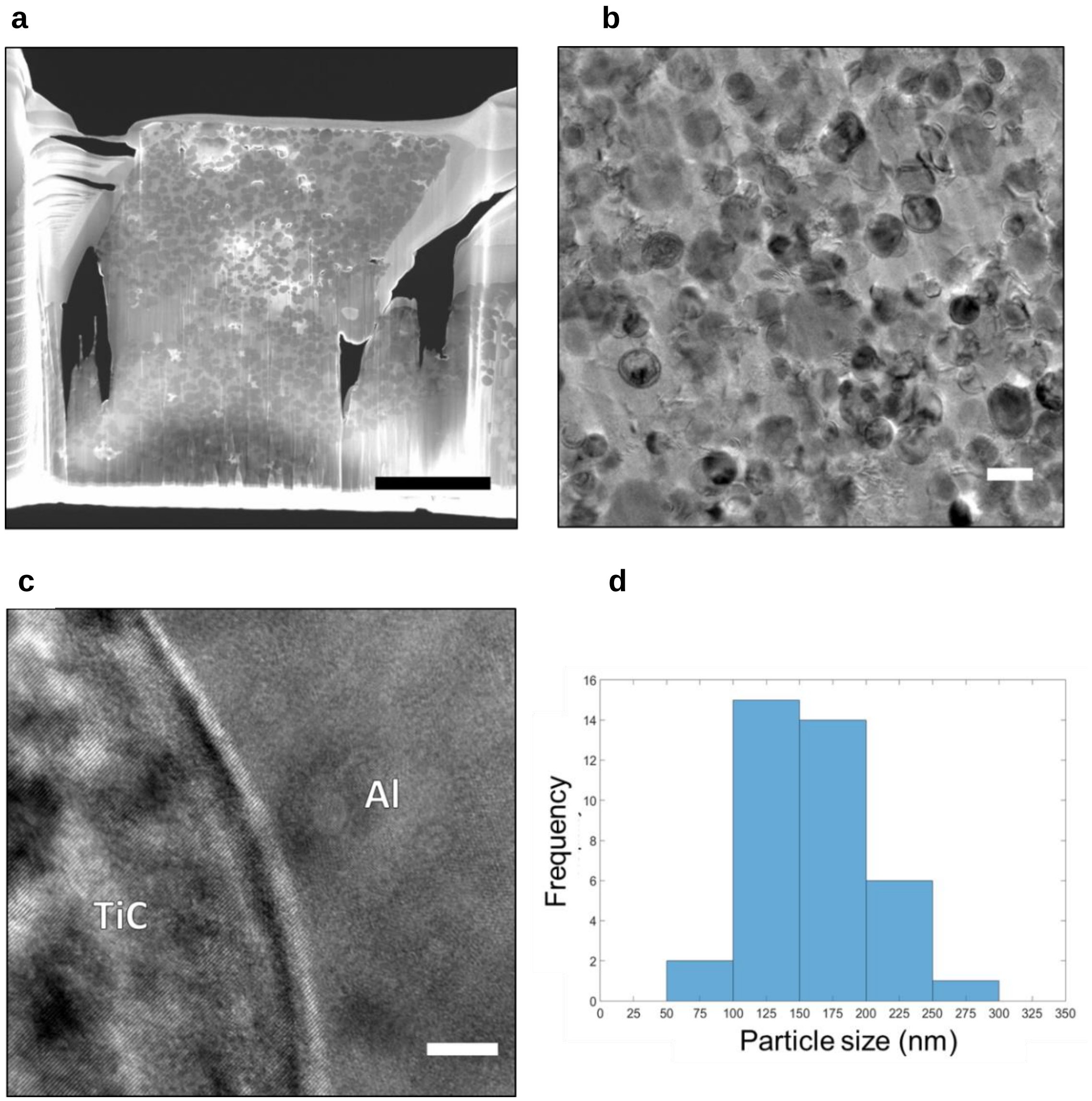

Supplementary Figure 4 | Nanostructure characterization of AMNC from compressed specimen. **a**, A thin-slice laser-deposited AMNC (35 vol.% TiC) specimen prepared by FIB. The specimen with a thickness of less than 100 nm was directly cut from a micropillar after the compression test. **b**, A bright-field TEM image indicates that TiC nanoparticles are well dispersed in Al matrix. **c**, A magnified image from **b**, reveals that TiC is bonded well with Al. A high resolution FFT filtered image can be found in

main body in Fig. 2d. **d**, A histogram of TiC nanoparticle size distribution analyzed from image b. Scale bar, 2 µm in a, 200 nm in b and 5 nm in c.

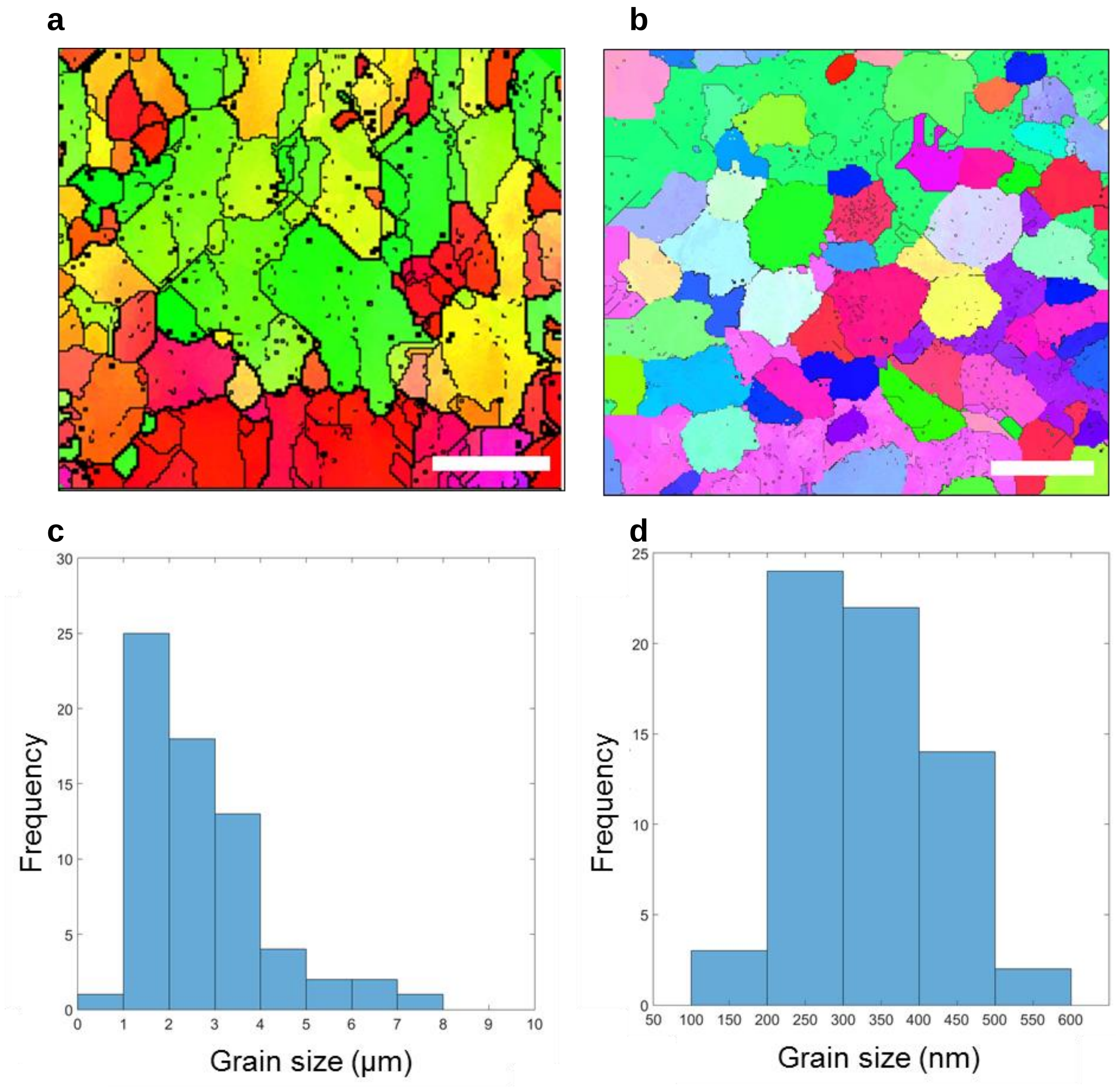

Supplementary Figure 5 | Grain maps of laser-deposited a, pure aluminum and **b**, AMNC (35 vol.% TiC). To clearly determine the grain size, we first show the Al phase only to eliminate the image processing error due to the dense TiC nanoparticles. The image reveals the grains by removing the nanoparticle phase. The distribution in grain size for the **c**, pure aluminum and **d**, AMNC (35 vol.% TiC, preheated at 300 °C). The average grain size of pure aluminum and AMNC (35 vol.% TiC) are about 2.7 ± 1.4μm and 331 ± 95 nm, respectively. Scale bar, 5 μm in a, and 500 nm in b.

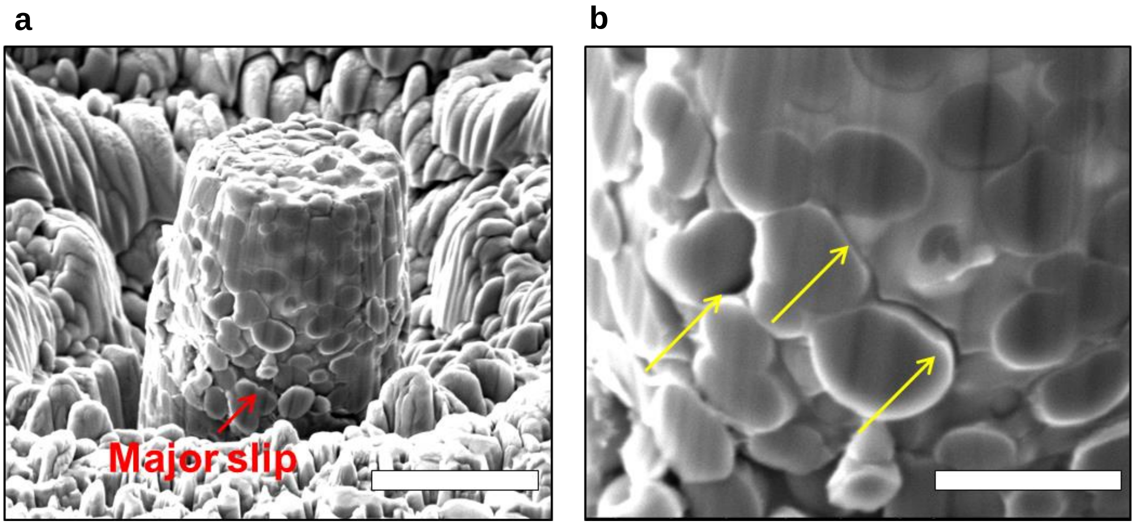

Supplementary Figure 6 | SEM images of a, compressed pillar showing the morphology of nanoparticles after bearing the load; **b**, A magnified image from **a.** The pillar presented in this figure is the same one tested in the in-situ microcompression movie. The nanoparticles are still able to bear the increasing load (starting around 34 seconds of the movie) even after the major slip band. No obvious slip traces were further observed till the end of the movie. Instead, the traces (yellow arrows) between nanoparticles and matrix appear gradually as the load increases continuously, showing the evidence that the nanoparticles bear the load during the deformation stage (Also see supplementary movie from 34s to 50s). Scale bar, 3 µm in a, and 1µm in b.

a

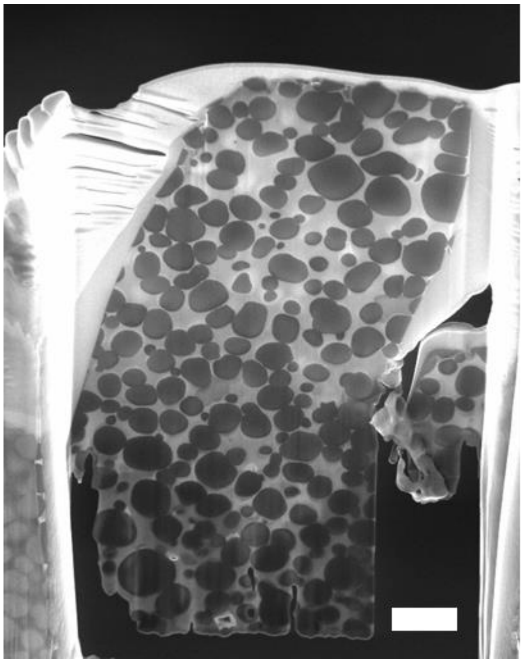

b

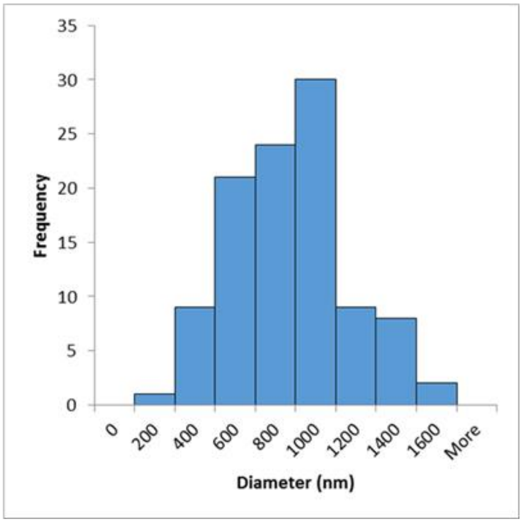

Supplementary Figure 7 | Microstructure characterization of AMNC (after 400 ^{o}C heat treatment) from compressed specimen. a, A thin-slice laser-deposited AMNC (35 vol.% TiC) specimen prepared by FIB (please note that this is high magnified image showing overlapping nanoparticles due to the transmission nature). **b**, a particle size distribution histogram analyzed from a. The average diameter is 783±295 nm corresponding to 77 MPa of Orowan strengthening. The Hall-Petch strengthening is estimated to 25 MPa, i.e., 10% contribution of the total strength. Scale bar, 1 μm in a.

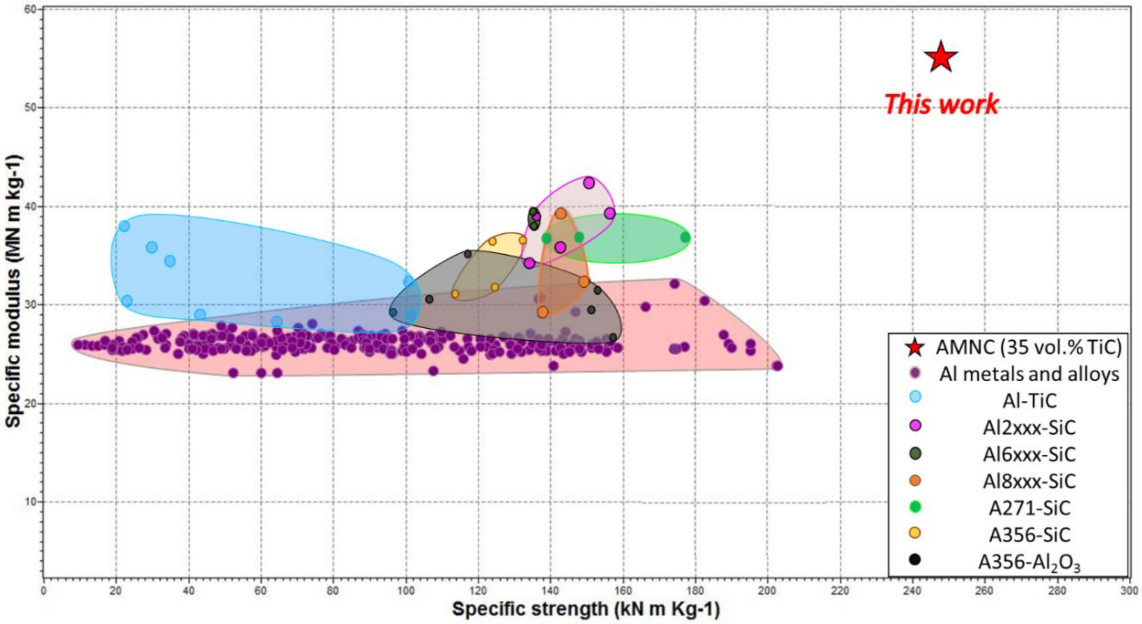

Supplementary Figure 8 | Comparison in specific Young's modulus and specific yield strength of AMNC (this work), Al alloys, and Al composites (plotted via CES Selector software, Granta Design, Cambridge, UK). Our AMNC clearly offers the highest specific modulus and yield strength among all aluminum materials.

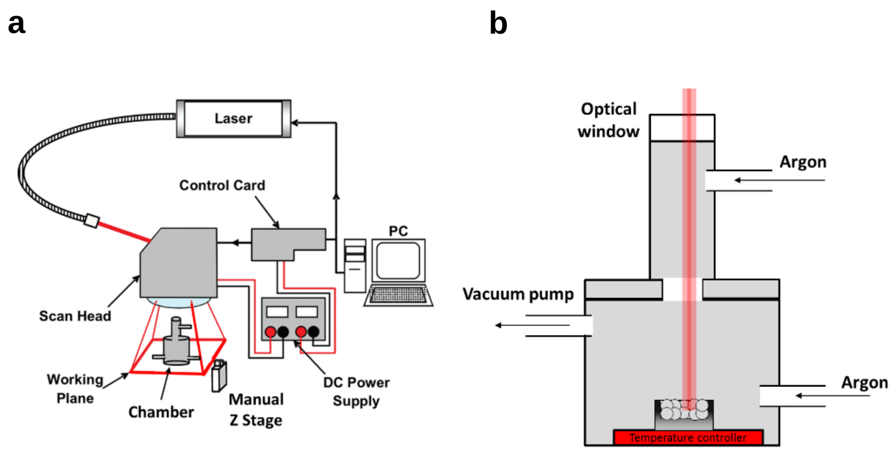

Supplementary Figure 9 | Schematic of lab-built laser additive manufacturing system. a, A 1070 nm fiber laser (SP-200C-W-S6-A-B, SPI Lasers) with a power of 200 W for laser deposition. A manual z-axis stage was used to accommodate the laser focal point. **b**, The vacuum chamber with a temperature control system for powder layers.

Supplementary Table 1

Authors	Fabrication method	Material system	Yield strength (MPa)	Elastic modulus (GPa)	Volume (%)	Weight (%)
Kennedy[2]	Casting	Al/TiC	63	89	10	-
Selcuk[3]	Casting	Al/TiC (T6)	301	84	7	10
Bauri[4]	Casting	Al/TiC	88,103,123	-	-	5
Kumar[5]	Casting	AlCu/TiC	87	-	-	10
Mazaheri[6]	Casting	Al/TiC	66	-	10	-
Shyu[7]	Casting	Al/TiC	296	94	-	-
Rai[8]	Casting	Al/TiC	80, 90, 100 (Hot rolled)	-	-	10
Wang[9]	Combustion synthesis	2009Al/TiC	120, 191, 248, 198	-	10, 15, 20, 30	-
Contreras[10]	Presureless Infiltration	Al/TiC	-	170	56	-
Mohapatra[11]	Hot Consolidation	Al/TiC	107, 180, 205	89 (20 vol.%)	5,10, 20	-

Table 1 summarizes the material properties of aluminum reinforced by TiC micro particles (Al-TiC composites) reported in literatures. Problems such as porosity, chemical reaction, poor wettability, poor incorporation, nonhomogeneous particle dispersion and distribution in the matrix were often observed, especially with reinforcements of smaller sizes[6, 12, 13]. It should be noted that even with 56 vol.% TiC micro powders incorporated into Al matrix using a pressureless infiltration technique, the elastic modulus of the Al-TiC composite is still only 170 GPa without any yield strength data[10]. It clearly shows that all Al-TiC composites offer much lower properties and Young's modulus than our AMNCs (with up to 35 Vol.% TiC nanoparticles in our study), which delivered a yield strength of up to 1000 MPa, plasticity over 10%, and Young's modulus of approximately 200 GPa.

Supplementary References

1. Tolochko Nikolay K. Absorptance of powder materials suitable for laser sintering. *Rapid Prototyping Journal* **6**, 155-161 (2000).

2. Kennedy AR, Karantzalis AE, Wyatt SM. The microstructure and mechanical properties of TiC and TiB2-reinforced cast metal matrix composites. *Journal of Materials Science* **34**, 933-940 (1999).

3. Selcuk C, Kennedy A. *Al–TiC composite made by the addition of master alloys pellets synthesised from reacted elemental powders* (2006).

4. Bauri R, Yadav D, Suhas G. Effect of friction stir processing (FSP) on microstructure and properties of Al–TiC in situ composite. *Materials Science and Engineering: A* **528**, 4732-4739 (2011).

5. Kumar A, Mahapatra MM, Jha PK. Fabrication and characterizations of mechanical properties of Al-4.5% Cu/10TiC composite by in-situ method. *Journal of Minerals and Materials Characterization and Engineering* **11**, 1075 (2012).

6. Mazaheri Y, Meratian M, Emadi R, Najarian AR. Comparison of microstructural and mechanical properties of Al–TiC, Al–B4C and Al–TiC–B4C composites prepared by casting techniques. *Materials Science and Engineering: A* **560**, 278-287 (2013).

7. Shyu RF, Ho CT. In situ reacted titanium carbide-reinforced aluminum alloys composite. *Journal of Materials Processing Technology* **171**, 411-416 (2006).

8. Rai RN, Rao AKP, Dutta GL, Chakraborty M. Forming Behaviour of Al-TiC In Situ Composites. *Materials Science Forum* **765**, 418-422 (2013).

9. Wang L*, et al.* Microstructure and tensile properties of in situ synthesized nano-sized TiCx/2009Al composites. *Materials & Design* **79**, 68-72 (2015).

10. Contreras A, Albiter A, Pérez R. Microstructural properties of the Al–Mgx/TiC composites obtained by infiltration techniques. *Journal of Physics: Condensed Matter* **16**, S2241-S2249 (2004).

11. Mohapatra S, Chaubey AK, Mishra DK, Singh SK. Fabrication of Al–TiC composites by hot consolidation technique: its microstructure and mechanical properties. *Journal of Materials Research and Technology* **5**, 117-122 (2016).

12. Karantzalis A, Lekatou A, Georgatis M, Poulas V, Mavros H. *Casting-Based Production of Al-TiC-AlB2 Composite Material Through the Use of KBF4 Salt* (2010).

13. Suthar J, Patel KM. Processing issues, machining, and applications of aluminum metal matrix composites. *Materials and Manufacturing Processes* **33**, 499-527 (2018).
