# Supplementary material for: Aluminum with dispersed nanoparticles by laser additive manufacturing
Source: Nat Commun. 2019 Sep 11;10:4124. doi: 10.1038/s41467-019-12047-2 (PMC6739343; doi:10.1038/s41467-019-12047-2)
Supplement: Supplementary file 2 — Description of Additional Supplementary Files [file 41467_2019_12047_MOESM2_ESM.docx]

Description of Additional Supplementary Files

**Supplementary Movie 1:** In-situ microcompression at 400 °C
